# Contrast enhanced magnetic resonance angiography in children: initial experience at 3.0 Tesla

**DOI:** 10.1186/1532-429X-15-S1-W36

**Published:** 2013-01-30

**Authors:** SN Khan, C Meehan, A Plotnik, I Ayad, S Patel, I Boechat, P Finn

**Affiliations:** 1 Radiology, UCLA, Los Angeles, CA, USA; 2 Anesthesia, UCLA, Los Angeles, CA, USA

## Background

To assess the role of contrast enhanced magnetic resonance angiography (CEMRA) at 3.0T in pediatric patients referred for vascular evaluation, and to compare the technical and diagnostic performance of a clinically similar control group at 1.5T.

## Methods

Fifty pediatric patients referred for vascular evaluation and without evidence of congenital heart disease, were evaluated with CEMRA. Thirty-five patients received 37 studies at 3.0T (age 0.4 -16.5 years, mean 5.8 ± 4.7 years. Fifteen patients received 16 studies at 1.5T (age 0.1 - 17.5 years, mean 5.8 ± 6.4 years). CEMRA was performed in three phases: arterial, early venous and late venous. Two independent observers analyzed the studies for image quality, artifacts and vessel definition.

## Results

Overall image quality and vessel definition scores were higher at 3.0T than 1.5T in the arterial and early venous phase, however not the late venous phase. Overall diagnostic performance was comparable at both field strengths. Breathing, pulsation and parallel acquisition artifact was found to some extent in all phases, but ventilated patients received significantly higher image quality scores and vessel definition scores than conscious patients.

**Figure 1:**
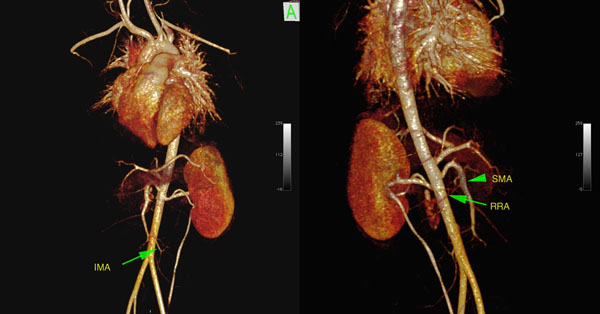
9 year old male with right vestigial kidney, contrast enhanced MRA performed at 3.0T. Left, 3D volume rendered reconstruction, anterior view, IMA = inferior mesenteric artery. Right, 3D volume rendered reconstruction, posterior view, SMA = superior mesenteric artery, RRA = right renal artery.

## Conclusions

CEMRA at 3.0T and 1.5T produces diagnostic quality studies for pre-and post-transplantation vascular assessment in pediatric patients. For optimum image quality and spatial resolution, the use of 3.0T MRA with controlled ventilation is recommended if readily available.

## Funding

Siemens Research Grant

